# Mode of action, chemistry and defensive efficacy of the osmeterium in the caterpillar *Battus polydamas archidamas*

**DOI:** 10.1038/s41598-023-33390-x

**Published:** 2023-04-24

**Authors:** Valeria Palma-Onetto, Jan Bergmann, Marcia González-Teuber

**Affiliations:** 1 Departamento de Química Ambiental, Facultad de Ciencias, Universidad Católica de La Santísima Concepción, Concepción, Chile; 2 Instituto de Química, Facultad de Ciencias, Pontificia Universidad Católica de Valparaíso, Valparaíso, Chile; 3 Departamento de Genética Molecular y Microbiología, Facultad de Ciencias Biológicas, Pontificia Universidad Católica de Chile, Santiago, Chile

**Keywords:** Cell biology, Chemical biology, Ecology, Physiology, Zoology

## Abstract

Chemical secretions are one of the main defensive mechanisms in insects. The osmeterium is a unique organ in larvae of Papilionidae (Lepidoptera), which is everted upon disturbance, secreting odoriferous volatiles. Here, using larvae of the specialized butterfly *Battus polydamas archidamas* (Papilionidae: Troidini), we aimed to understand the mode of action of the osmeterium, the chemical composition and origin of the secretion, as well as its defensive efficiency against a natural predator. We described osmeterium’s morphology, ultramorphology, structure, ultrastructure, and chemistry. Additionally, behavioral assays of the osmeterial secretion against a predator were developed. We showed that the osmeterium is composed of tubular arms (made up by epidermal cells) and of two ellipsoid glands, which possess a secretory function. The eversion and retraction of the osmeterium are dependent on the internal pressure generated by the hemolymph, and by longitudinal muscles that connect the abdomen with the apex of the osmeterium. Germacrene A was the main compound present in the secretion. Minor monoterpenes (sabinene and ß-pinene) and sesquiterpenes ((E)-β-caryophyllene, selina-3,7(11)-diene, and other some unidentified compounds) were also detected. Only sesquiterpenes (with the exception of (E)-β-caryophyllene) are likely to be synthesized in the osmeterium-associated glands. Furthermore, the osmeterial secretion proved to deter predatory ants. Our results suggest that the osmeterium, besides serving as an aposematic warning for enemies, is an efficient chemical defense, with its own synthesis of irritant volatiles.

Adults and immature stages of insects often face intense predation. To overcome this predation, insects employ numerous defensive strategies, including passive mechanisms such as gregariousness or a cryptic appearance; and active and energetically expensive defensive strategies, such as escaping, postural defenses, fighting, and chemical defenses^1–3^. Chemical defenses are highlighted to be one of the most widely and effective defensive mechanisms among insects^4–7^. For Lepidoptera larvae, secretory exocrine glands, regurgitated plant extracts, de novo production of chemical defenses, and sequestration of toxic compounds from host plants are the main chemical defenses used against predators^5,8–10^.

Larvae of the family Papilionidae (Lepidoptera) possess an osmeterium as a central part of their defensive mechanisms. The osmeterium is a Y-shaped eversible organ located dorsally just behind the head^11,12^. It possesses two exocrine glands at the base of each arm^5,13,14^. Its color is usually yellow/orange, acting as an aposematic warning of both sight and smell^15^. When undisturbed, the osmeterium remains retracted inside the larval body. Upon disturbance, however, it is filled with hemolymph and is everted, and defensive chemicals are released^5,11,16^ (Fig. 1 and Supplementary Video S1). Specialized muscles are likely taking part in the subsequent retraction process of the osmeterium^17^. However, the specific mode of action as well as the structures involved in the eversion and retraction processes of the osmeterium are still not fully understood.

**Figure 1:**
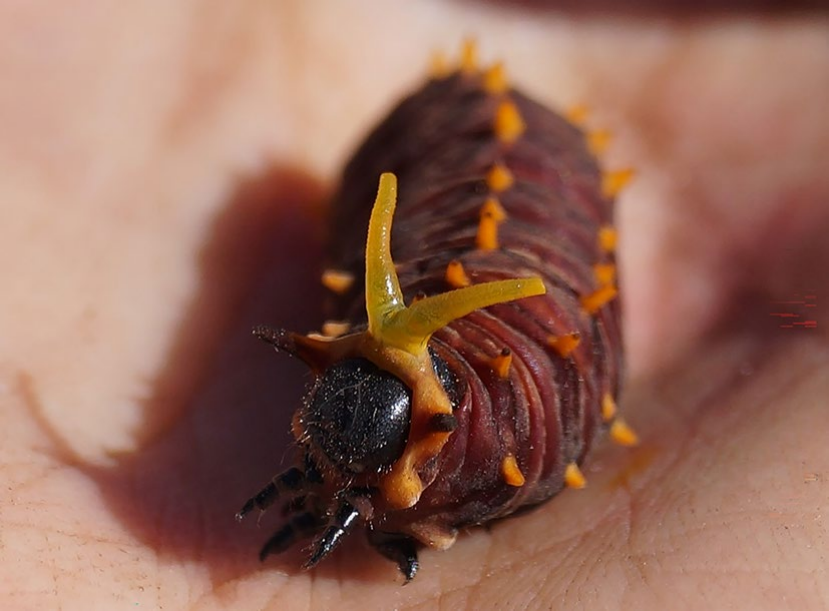
Larval individual of *Battus polydamas archidamas*, fully exposing its osmeterium.

The osmeterial secretion of Papilionidae larvae comprises a mixture of volatile organic compounds (VOCs) with an unpleasant odor. The chemical composition has been studied in about 20 species, and several compounds have been identified to be part of the secretions, including mono- and sesquiterpenes, aliphatic short-chain acids, and esters^11,12,18–28^. In general, the osmeterium’s defensive role appears to be more effective against invertebrate predators, including ants and praying mantids^18,28–30^, than against vertebrates^25,31^. Honda^21–23^ observed in different papilionids that monoterpenes showed a considerable toxic effect against ants, whereas most aliphatic acids exerted weak toxicity against them. The origin of the osmeterium odor has been linked to volatiles released by exocrine glands^14^. Indeed, numerous compounds such as terpenes, acetic acid, and fatty acid derivatives seem to be synthesized by the larva itself^24–26^. Nevertheless, some of them, such as mono and sesquiterpenes and non-volatile compounds, including aristolochic acids, are sequestered from the host plant^11,24,32^, and can be present in the osmeterium as a by-product of the hemolymph. Thus, the origin of osmeterium’s volatile compounds is unclear: they are either synthesized in the osmoterium itself or are a hemolymph by-product.

*Battus polydamas archidamas* (Linnaeus, 1758) (Papilionidae: Troidini) is the only Papilionidae species present in Chile. Its larvae are dark burgundy in color and are known for everting its orange osmeterium upon disturbance^12^. Larvae of this species are aposematic and feed exclusively on plants of the genus *Aristolochia*, which contain toxic compounds that include benzylisoquinoline alkaloids and aristolochic acids^33,34^. Larvae of *B. polydamas archidamas* are well known for their ability to sequester toxic compounds (i.e., aristolochic acids) from the host plant, which are stored in the hemolymph and other parts of the body^32^. In this study, a multidisciplinary approach is used to understand the mode of action of the osmeterium, the chemical composition of the secretion, the biosynthetic origin of secreted VOCs, and its defensive role against a natural predator. Using *B. polydamas archidamas*, this study contributes to knowledge about caterpillar defense strategies by describing the morphology, ultramorphology, structure, ultrastructure, chemistry, and ecological role of the osmeterium.

## Results

### Morphology

**Figure 2:**
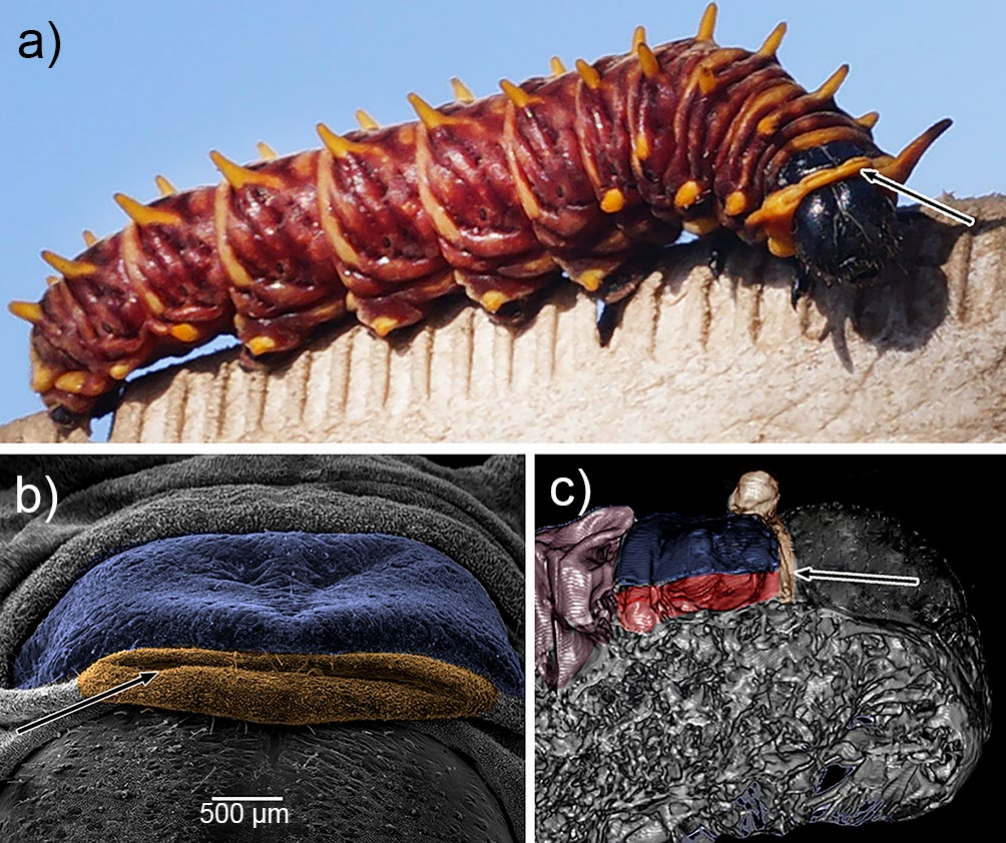
Osmeterium location in *Battus polydamas archidamas* when it is retracted. (**a**) alive larva; (**b**) prothorax view under scanning electron microscope; note the prothorax represented in blue and the osmeterium in orange; (**c**) prothorax and head of larvae under micro-CT; note the prothorax represented in blue and the space where most part of the osmeterium is saved in red.

**Figure 3:**
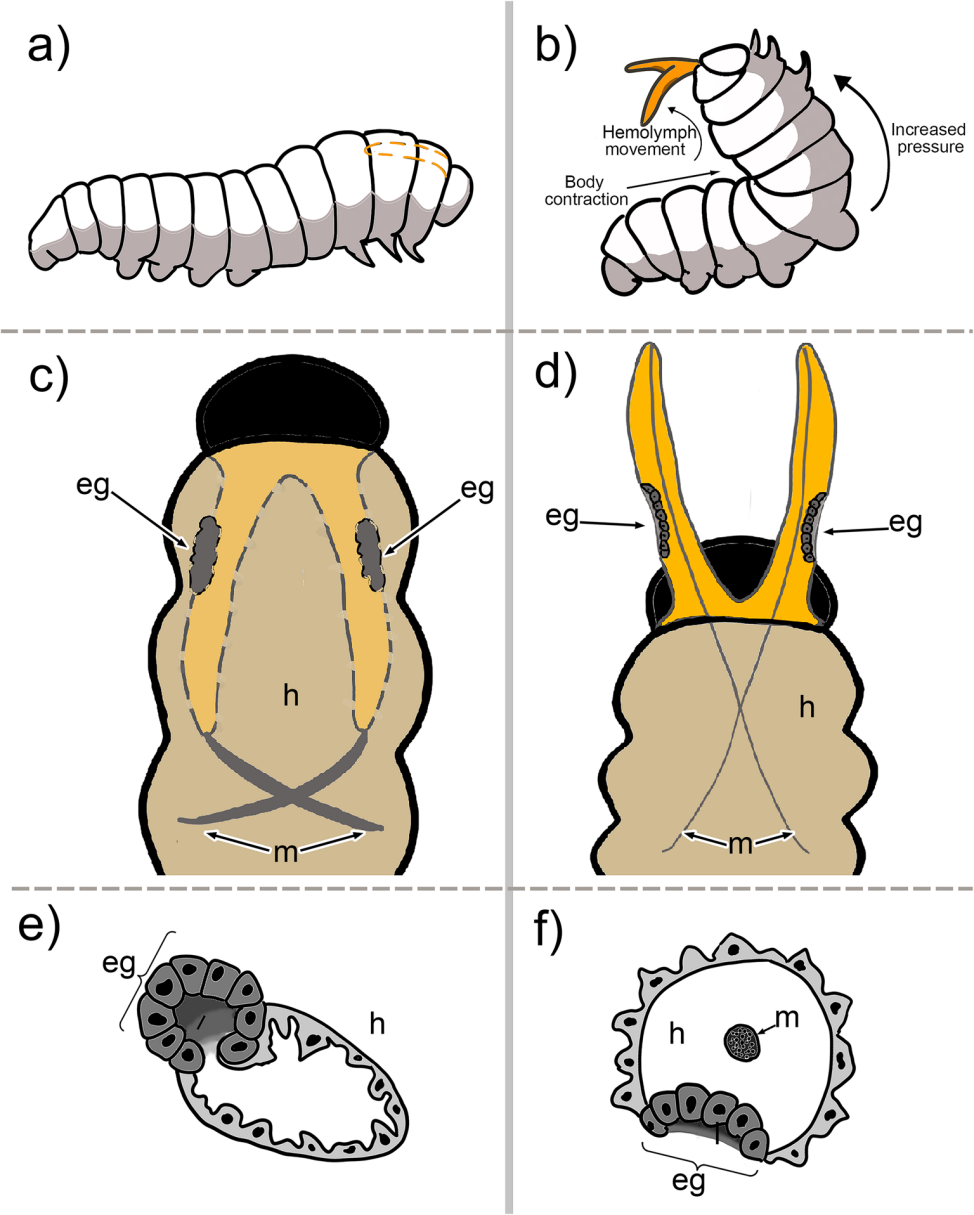
Diagram illustrating the mechanisms of eversion and retraction of the osmeterium in *Battus polydamas archidamas*. From the top to the bottom: general location of the osmeterium in the caterpillar (**a**) and (**b**); specific location of the osmeterium, ellipsoid glands and associated muscles (**c**) and (**d**); and a cross section of one arm of the osmeterium (**e**) and (**f**). From the left to the right, illustrations represent the retracted osmeterium (**a**, **c**, and **e**) and the everted osmeterium (**b**, **d** and **f**), respectively. Abbreviations: eg, ellipsoid glands; h, hemolymph; m, muscle.

**Figure 4:**
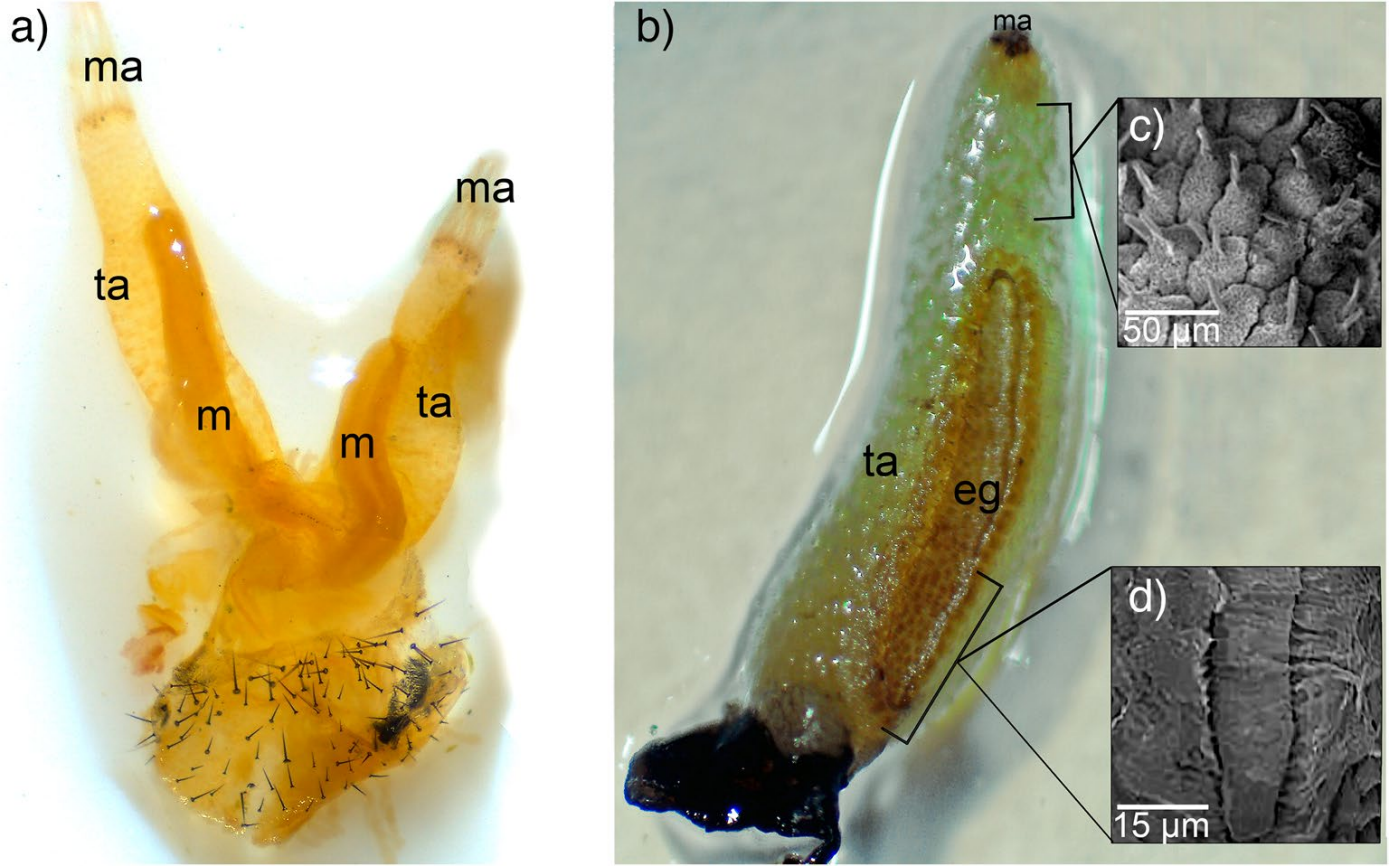
Morphology and ultramorphology of the osmeterium in *Battus polydamas archidamas*. (**a**) General view of a dissection of the osmeterium, note the muscles running inside of the osmeterium, which were torn from the larval body and the osmeterium apex. (**b**) One arm of the osmeterium that shows two sectors composing it. Scanning electron microscope detailed images of the tubular arm (**c**) and the ellipsoid gland (**d**). Abbreviations: eg, ellipsoid gland; m, muscle; ma, attachment of the osmeterium to the muscle; ta, tubular arm.

When retracted, the osmeterium was located at the dorsal part of the body. The base and part of the arms were kept inside the prothorax within a dorsal cavity, which opens at the dorsal junction between head and thorax, allowing it to be everted when needed (Figs. 2 and 3). Although a main part of the retracted osmeterium is situated under the prothorax shield, the arms were observed to reach the beginning of the third segment of the thorax (T3) (Fig. 3). Once everted, the osmeterium was Y-shaped, consisting of two arms branching from a common thick base (Figs. 1, 3 and 4).

### Structure

Exerting manual pressure on the larval abdomen immediately resulted in the eversion of the osmeterium. The osmeterium had an orange color as well as the hemolymph circulating inside the larval body (Figs. 1 and 4a). The osmeterium is composed of two different structures: the tubular arms and the ellipsoid glands (Figs. 3 and 4b). The glands are not visible when the osmeterium is fully everted. Nevertheless, these are visible once the hemolymph is removed, revealing a pairwise ellipsoid structure that is located at the base of the posterior face of each arm, where the bifurcation of each tubular arm begins (Figs. 3 and 4b). The gland possesses well developed cells, which forms a convex yellow area, darker than the rest of the osmeterium. The ellipsoid gland can be up to 2 mm long; in a slightly deflated osmeterium the ellipsoid gland can be about three-quarters the size of the osmeterium (Fig. 4b), whereas in a fully protruded osmeterium, it reaches about half the size. The surface of both tubular arms showed squamous structures with a single papilla (Fig. 4c), while the ellipsoid gland presented a pore-like appearance with folded, very irregular structures around (Fig. 4d). Longitudinal muscles ran from the larval abdomen (A1) to the apex of the osmeterium (Fig. 3 and Supplementary Fig. S1). The contraction of dorsal muscles increased the hydrostatic pressure inside the larva, which causes osmeterium’s eversion, and consequently the passive stretch of elastic longitudinal muscles at its apex (Fig. 3). A cross-section of the retracted osmeterium showed epidermal cells of tubular arms and rounded ellipsoid glands looking inwards as well as its lumen (Fig. 3e and Supplementary Fig. S1). On the other hand, a cross-section of the everted osmeterium showed ordered ellipsoid glands exposed to the outside, as well as its lumen, and muscle cells running throughout the osmeterium. Thus, the ellipsoid glands, which were folded in the contracted osmeterium, are exposed when it is everted (Fig. 3f and Supplementary Fig. S1).

### Ultrastructure: epidermal cells

**Figure 5:**
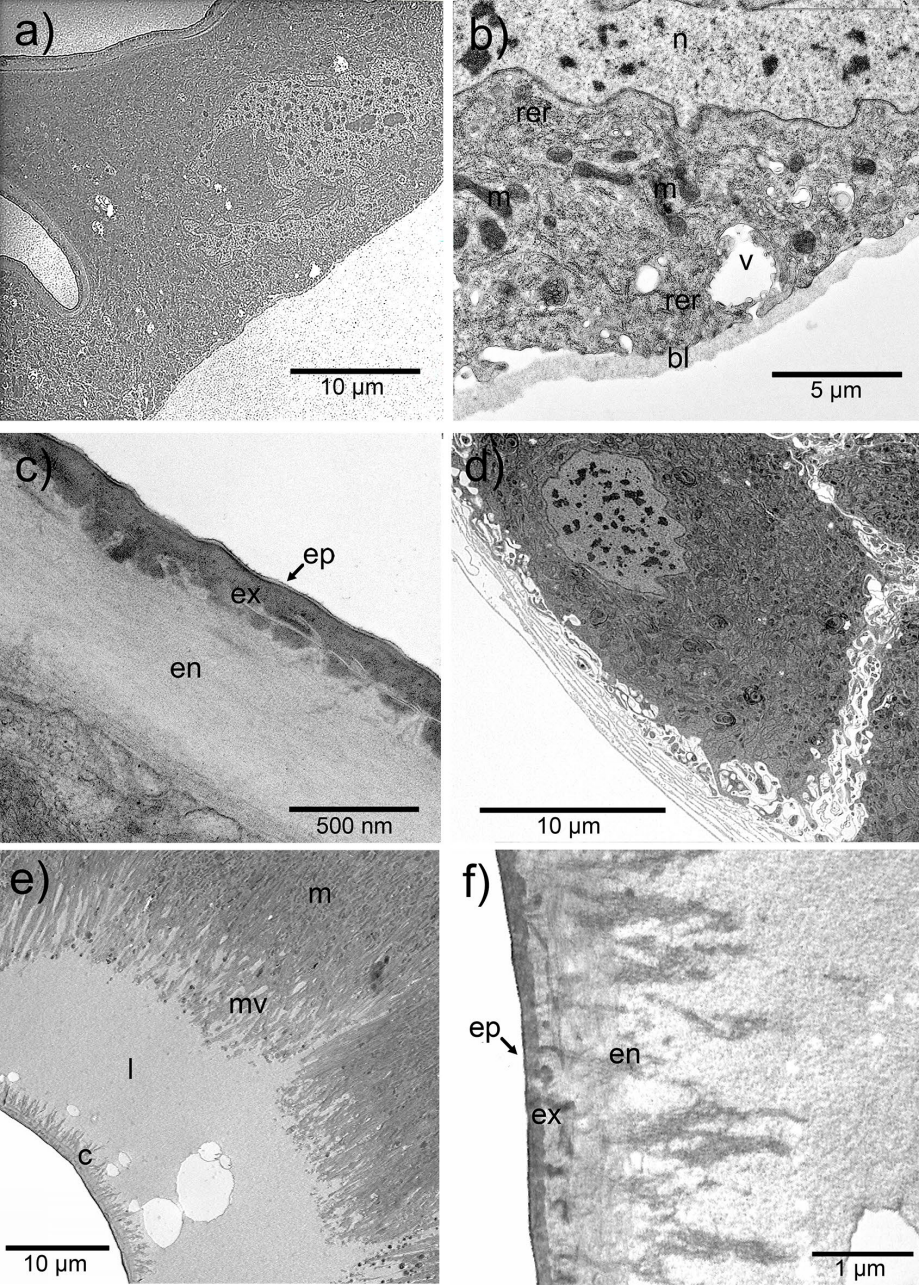
Ultrastructure of cells that compose the osmeterium in *Battus polydamas archidamas.* (**a**) Overall development of epidermal cells. (**b**) Detail of the cytoplasm of epidermal cell. (**c**) Well-defined cuticle underlying the epidermal cells. (**d**) Overall development of the ellipsoid glands. Note the extracellular channels running up into the cell. (**e**) Apex of the ellipsoid gland cells that shows abundant microvilli and dense secretion-like particles at the lumen of the gland. (**f**) Cuticle at the middle part of the gland pore that shows highly modified endo and exocuticle. Abbreviations: bl, basement lamella; c, cuticle; en, endocuticle; ep, epicuticle; ex, exocuticle; l, lumen; m, mitochondria; mv, microvilli; n, nucleus; rer, rough endoplasmic reticulum; v, vesicle.

Cells in the tubular arms can be characterized as epidermal cells, without any feature that may indicate a specific secretory activity (Fig. 5). These cells were irregularly shaped, sometimes semi-rounded or triangular-like, of about 10 to 25 µm long and about 30 µm wide, with granular cytoplasm and semicircular, well-developed nuclei with elongated oval shape and about 10 µm diameter (Fig. 5a). Cells were packed closely together and linked by septate desmosomes. The plasma membrane adjacent to the hemolymph bears a basement lamella of 200–300 nm thick (Fig. 5b). The cytoplasm contained numerous ribosomes, most of which were attached to the rough endoplasmic reticulum. Mitochondria were about 1 µm long, elongate with predominantly transverse cristae. Although not so frequent, some vesicles were visible, especially near the hemolymph. Epidermal cells adjoin with a well-formed, although thin, cuticle (about 1 µm thick), which is made up of three layers: an endocuticle of helicoid structure (760 nm), an exocuticle (300 nm, without discernible sublayers), and a thin epicuticle (10 nm) (Fig. 5c).

### Ultrastructure: ellipsoid glands

Cells in the glandular section can be characterized as secretory, and could easily be differentiated from non-secretory epidermal cells of tubular arms (Fig. 3e,f and Supplementary Fig. S1). Glands were predominantly made up of oval or columnar class 1 secretory cells (according to^35^). Glands were about 50–80 µm long and 40–50 µm wide each, and were projected into the hemolymph of tubular arms of the retracted osmeterium. The basal area of the cells was covered by a basement lamella of 4 to 6 sheets up to 100 nm thick, beneath which the plasma membrane was thrown into extensive infoldings creating extracellular channels running up into the cell (Fig. 5d). On the other side, the apex of the cell showed abundant thin microvilli (up to 10 µm length and 150 nm thick) protruded to the gland lumen (Fig. 5e). The ellipsoid gland lacks a reservoir; thus, secretions are apparently stored in the space between the secretory epithelium and the modified cuticle (Fig. 5e). The cytoplasm showed a high amount of smooth endoplasmic reticulum (ER), abundant ribosomes, vesicles of different electron densities, abundant mitochondria, glycogen granules, myelin figures and sparse rough ER, the latter being mainly located around the nucleus. Ribosomes were randomly dispersed through the cytoplasm, where they were mainly attached to the cisternae of the ER at the base of the cell (Fig. 5a). Low-density vesicles were especially common and were frequently observed in the microvilli, apparently to release its content at the cell apex through the microvilli (Fig. 5e). Occasional electron-dense vesicles were also present in the microvilli (Fig. 5e). Electron-dense vesicles located in the cytoplasm and containing differentiated structures, were present in the cells, and are interpreted as components of the lysosome-cytolysome vacuolar apparatus. The nuclei of glandular cells were slightly irregular in profile, and have a maximum size of about 30 µm, with decondensed chromatin (Fig. 5a). Mitochondria were small (0.25 to 1 µm) and highly abundant, especially at the apex of the cell, near the microvilli. Many tracheoles pass through the intercellular space and basement lamella. The cuticle that covers ellipsoid glands was thin and slightly modified at the borders, composed by a well-structured endocuticle (0.86–1 µm thick), a thin exocuticle with channels running through it (270–300 nm thick), an inner epicuticle about 14 nm thick, and an outer epicuticle about 8–9 nm thick. The gland pore (middle part of the gland) presented a highly modified cuticle, with an irregular lamellate endocuticle (2.5–2.7 µm thick, with deposits of electron-dense material without resolved structure), a thin exocuticle of about 160–375 nm thick (with few and not well demarked cavities), and a thin epicuticle of 25 nm thick (Fig. 5f). Lumen at the ellipsoid gland borders was clean and contained several vesicles, which sometimes seem to fade away when in contact with the cuticle. The lumen of the gland pore (15–22 µm thick) showed a slightly dense homogeneous content with few large vacuoles. It was internally delimited by the thin cuticular layer (Fig. 5e), which corresponded to the pore opening-like of the externally visible gland (Fig. 4d). Additionally, big irregularly rounded electron-lucent cavities (up to 6 µm in diameter) were present in the lumen, near the cuticle (Fig. 5e). For detailed representations of the ultrastructure of the ellipsoid gland, see Supplementary Fig. S2.

### Chemical composition

**Figure 6:**
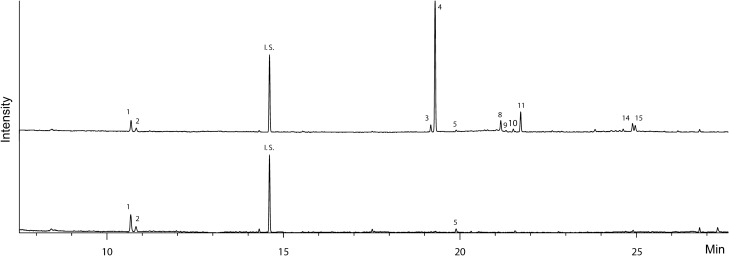
Comparison of the gas chromatogram of extracts of the osmeterial secretion (up) against the hemolymph of *Battus polydamas archidamas* (down). I.S. = internal standard (1-bromooctane). VOC identification is shown in Table 1.

Analysis by Gas Chromatography-Mass Spectrometry (GC/MS) revealed the presence of several compounds in the osmeterial secretion that were tentatively identified as monoterpenes (peaks 1 and 2, Table 1) and sesquiterpenes (peaks 3–15, Table 1), on the basis of their retention indices and mass spectra. By comparison with authentic standards, peaks 1 and 2 were readily identified as sabinene and β-pinene, respectively. The mass spectra of peaks 3 and 4 suggested them to be cis-and trans-isomers of β-elemene, which was corroborated by comparison with an authentic standard. The broad peak between ca. 19.5 and 21 min (retention time) suggested the presence of a compound that is unstable under the conditions of analysis. It is known that the sesquiterpene germacrene A easily undergoes Cope rearrangement to form β-elemene^36^, which led us to believe that the β-elemene in the sample was an artefact formed from germacrene A. Analysis of the extract at a lower injector temperature (100 °C) indeed led to the complete disappearance of peaks 3 and 4, and an increase in the relative amount of peak 9, as well as the before mentioned broad peak (Supplementary Fig. S3). The mass spectrum of this compound and its retention index on the polar Stabilwax column were in good agreement with those reported for germacrene A^23^, supporting our conclusion. Further proof was obtained by catalytic hydrogenation of the extract and subsequent analysis by GC/MS. After hydrogenation, the major compounds present were germacranes (ca. 85%), together with small amounts of selinanes (10%), eremophilane (4%), and elemane (1%). The identities of the saturated compounds were corroborated by comparison of the retention indices and mass spectra of the hydrogenation products of germacrene B (for germacranes), the essential oil of celery seeds (for selinanes), valencene (for eremophilane), and β-elemene (for elemane). All this information together strongly indicated that the major compound originally present in the osmeterial secretion is germacrene A. Peak 5 was identified as (E)-β-caryophyllene by comparison with an authentic standard. Peaks 6 and 7 were tentatively assigned as β-selinene and α-selinene, respectively, on the basis of their retention indices and mass spectra. The identification was confirmed by comparison with α- and β-selinene contained in celery seed essential oil^37^. Apparently, these compounds are derived from transformation of germacrene A, as they were not detected upon analysis using a lower injector temperature (Supplementary Fig. S3). The retention index and the mass spectrum of peak 10 were good matches to selina-3,7(11)-diene, but this tentative identification was not confirmed by comparison with a standard. Peaks 8 and 11 were not identified, but very likely are sesquiterpenes as well, as inferred from the general fragmentation pattern of their mass spectra. The mass spectrum of peak 8 shows a molecular ion at m/z 204, consistent with a molecular formula of C_15_H_24_, while peak 11 has a small but visible molecular ion at m/z 220, for which a molecular formula of C_15_H_24_O seems plausible. Similarly, peaks 12–15 were not identified, however, based on their mass spectra and molecular ions, they are most likely oxygenated sesquiterpenes with a molecular formula of C_15_H_24_O. In hexane extracts of the hemolymph only compounds 1, 2, and 5 were detected in roughly the same amount and proportion as in the osmeterial secretion (Fig. 6).

**Table 1:** Compounds present in the osmeterial secretion of *Battus polydamas archidamas*.

No	RI (SLB-5)	RI (Wax)	Compound	Relative abundance [%]^a^
1	975	n.d.^b^	sabinene	3
2	980	n.d	ß-pinene	< 1
3	1391	1570	*cis*-ß-elemene^c^	
4	1398	1580	*trans*-ß-elemene^c^	
5	1434	n.d	(*E*)-β-caryophyllene	< 1
6	1504	n.d	β-selinene^c^	
7	1510	n.d	α-selinene^c^	
8	1512	1712	C_15_H_24_	7
9	1522	1750	germacrene A	75
10	1536	n.d	selina-3,7(11)-diene	2
11	1549	1835	C_15_H_24_O	10
12	1693	n.d	C_15_H_24_O	< 1
13	1750	n.d	C_15_H_24_O	< 1
14	1770	2459	C_15_H_24_O	2
15	1776	2183	C_15_H_24_O	1

^a^Due to the chromatographic behavior of germacrene A, the relative abundance of all compounds was estimated considering data from analyses on the non-polar and polar columns, and from the hydrogenated samples. The abundance of compounds deemed artefacts from the transformation of germacrene A were added to the relative abundance of germacrene A. ^b^n.d. = not determined. ^c^These compounds are artefacts stemming from the transformation of germacrene A.

### Behavioral assays

The bioassays showed that workers of the ant *Linepithema humile* (Mayr, 1868) were significantly deterred by the osmeterial secretion (mean ± SE: 1.6 ± 0.3 ants) compared to the control (5.6 ± 0.4 ants) (W = 204, *p* < 0.001, Wilcoxon test). Once ants came into contact with the osmeterium extract, they began to vigorously clean themselves, and then quickly moved toward the control side. On the other hand, ants did not show a significant difference in reaction towards the osmeterium (5.4 ± 2.2 ants) and hemolymph (4.8 ± 2.3 ants) extracts (W = 72.5, *p* = 0.203). In this case, ants were observed to vigorously clean themselves at both sides of the Petri dish, curving their bodies to clean all parts of it with their legs and mouth.

## Discussion

The osmeterium of *B. polydamas archidamas* was composed of: i) tubular arms made up by epidermal cells, with an apparent structural function; and ii) columnar ellipsoid glands, located at the base of the dorsal side of each arm, which possess a secretory function. Our results agree with previous general descriptions of the osmeterium for other Papilionidae species, which also describe a glandular patch on the posterior side of each osmeterium arm^13,14,17,19^. Nevertheless, it is highly different from the description given for the osmeterium in *Zerynthia polyxena* (Papilionidae), in which the ellipsoid glands are opened almost at the apex of each arm and possess a reservoir^38^. The mechanisms of eversion and retraction of the osmeterium in *B. polydamas archidamas* is dependent on the hemolymph and the longitudinal muscles connecting the larva abdomen with the apex of the osmeterium. Upon disturbance, larvae curl up the body, increasing the hydrostatic pressure inside of it. Thereby, the dorsal musculature is contracted and the hemolymph pushes out the osmeterium against the prothorax cavity, which leads to its eversion and the passive stretch of the longitudinal muscles. When returning to its original position, the longitudinal muscles are contracted, leading to the retraction of the osmeterium.

Epidermal cells in the tubular arms lacked specific organelles or structures that may allow the secretion of compounds. Thus, vesicles and ribosomes present there might be part of the lysosome-cytolysome vacuolar apparatus, related to the proper maintenance of cells, such as the conformation of the external surface and osmeterium cuticle, and substitution of old cells by new ones^13,19,39,40^. Furthermore, Martinez et al.^14^ suggested that these vesicles likely relate to the coloration of the tubular arms in the osmeterium of *Papilio thoas*. On the other hand, cells in the ellipsoid glands showed organelles and structures related to secretory activity, which coincides with previous studies in other Papilionidae species, including *Graphium agamemnon agamemnon* and *Papilio thoas*, which described the role of the osmeterium in the synthesis of substances^13,14^. Tracheas and tracheoles were observed running through the space between the cytoplasm and the basement membrane of the ellipsoid gland cells. These tracheas could be a general feature of the ellipsoid gland, since they have also been observed in the osmeterium of *Papilio demoleus*^39^, although it remains to be studied in other species. The latter would provide the surrounding cells a large amount of energy supply for the mitochondria, allowing a robust secretory activity^39^. Cuticles were also distinct between both non-secretory and secretory regions. The well composed and flexible cuticle of the tubular arms is likely a morphological adaptation, which allows the right and fast eversion of the osmeterium as well as the exposure of the glandular pore^5,13^. In contrast, the highly modified cuticle of the ellipsoid glands exhibits several microchannels, which suggests that secreted compounds are released throughout the cuticle.

The osmeterial secretion of *B. polydamas archidamas* is composed of monoterpenes and sesquiterpenes. The monoterpenes sabinene and ß-pinene and the sesquiterpene (E)-β-caryophyllene were detected in both the osmeterium and the hemolymph, suggesting that the origin of these compounds is not the osmeterium itself and that they are likely a by-product of the hemolymph. These VOCs are toxic^11,41,42^, and have been previously reported in the osmeterium of more than 20 species from the tribu Papilionini^11,14,28^ and in two species from the tribu Zerynthiniini^11^. In addition, sabinene and ß-pinene constitute an important part of the volatiles present in *Aristolochia chilensis*^43^, the host plant of *B. polydamas archidamas*. In contrast to the monoterpenes, most sesquiterpenes (with the exception of (E)-β-caryophyllene) were detected exclusively in the osmeterium, and not in the hemolymph. This suggests that they are secreted by the specialized glands located in the osmeterium. Germacrene A, the main compound in the secretion of *B. polydamas archidamas*, is relatively widespread in nature, probably because it is an important intermediate in the biosynthesis of many sesquiterpenes of the eudesmane- and guaiane-type and structures derived thereof^44^. Furthermore, it is synthesized by defensive glands in numerous insects^45–48^ and it has been found in the osmeterial secretions of various Papilio species^11,20–23^. Germacrene A is an unstable compound that easily isomerizes to more stable compounds. The odd gas chromatographic behavior has been described before^20,49^ and is due to a thermally induced Cope rearrangement resulting in the formation of β-elemene, which takes place partly in the injector and partly in the column^20^. Acidic conditions induce cyclization reactions leading to the formation of α-selinene, β-selinene, and selina-4,11-diene as main products. In the presence of water, the intermediate carbocation formed from germacrene A can also react to give sesquiterpene alcohols such as selin-11-en-4α-ol or neointermedeol^50^. Furthermore, Eisner et al.^51^ reported the isolation of β-selinene and selin-11-en-4α-ol from the osmeterial secretion collected from a population of *Battus polydamas*. It remains unclear if these two compounds are artefacts from the transformation of germacrene A, or if there are differences between geographically distant populations of caterpillars.

Behavioral assays showed a deterrent effect of the osmeterial secretion of *B. polydamas archidamas* against the predator, *L. humile* ants. Previous studies have reported a toxic function for the osmeterium either when predators were exposed to the larvae fully displaying the osmeterium or when predators were fed with artificial diets containing single VOCs^25,28,52,53^. Here, we showed additional evidence that the osmeterium-associated chemistry has a direct defensive efficacy against the natural predator. Considering that the hemolymph showed also a deterrent effect against ants, our study suggests that *B. polydamas archidamas* combines different chemical defensive systems, including deterrent volatiles in the osmeterium with volatile and non-volatile toxic compounds (i.e., aristolochic acids^43^) present in the hemolymph. The combination of two defense lines (including toxic hemolymph) has been previously described for Coccinelidae larvae^54,55^, which may synergistically help insects to defend against a wide array of predators, including invertebrates and vertebrates.

This study used a multidisciplinary approach, including morphology, structure, chemistry and ecology to describe and understand the mode of action and function of the eversible defensive organ in a Papillionidae species. Our results lead us to conclude that the osmeterium, besides serving as an aposematic warning for potential enemies^15^, is an efficient chemical defense against natural predators, with its own synthesis of deterrent volatiles.

## Material and methods

### Study system

Third instar larvae of *B. polydamas archidamas* were collected from *Aristolochia chilensis* plants at Praderas de lo Aguirre (33°27′40.8″S 70°54′33.0″W) near Santiago, Chile. The site has a mean annual temperature of 15.3 °C, with maximums of 39 °C (own records) and receives an average of 77 mm of precipitation annually (data reported by the meteorological station: 855,740 (SCEL)). The internal observation of the larvae as well as observations under the microscope or x-ray microtomography contributed to samples’ deterioration. Voucher specimens, in which only osmeterium was removed, were preserved in 80% ethanol at Laboratory of Chemical Ecology at Universidad Católica de la Santísima Concepción (Concepción, Chile).

### Morphology: stereomicroscope and X-ray microtomography (MicroCT scanning)

To observe the muscular system associated with the osmeterium in *B. polydamas archidamas*, four larvae were killed by cold and dissected at the thorax and abdomen under a Leica® MZ7.5 stereomicroscope. To examine the internal space used by the osmeterium when it is retracted, as well as the muscles involved in the retraction and eversion processes, larvae were scanned in a Skyscan1272 high-resolution microtomographer (Bruker MicroCT, Kontich, Belgium), with a Hamamatsu L11871_20 X-ray source and a XIMEA xiRAY11 scan with a 9 µm pixel size. The following scanning parameters for larval scans were used: source voltage 65 kV, the source current 125 µA, and image rotation scan 360° with a 0.50° rotation step, and scanned in three steps, which were combined into a single volume with a scan duration of 16 min: 46 seg. A Bruker MicroCT Skyscan system software (NRecon, Dataviewer, CTAn) was used to join scanned steps into single 3D volume, and obtain the cross-sectional images (2D slices). Samples were not stained before use. 3D volumes were rendered using the software 3D slicer (version 4.11.2021)^56^. Color and light effects were created using Adobe Photoshop (2021).

### Ultramorphology: scanning electron microscopy

To superficially characterize the morphology of the cells that make up the osmeterium externally five larvae were cryoanesthetized at −4 °C for 5 min. Osmeteriums were then dissected in a saline solution (0.1 M NaCl + 0.1 M KH_2_PO_4_ + 0.1 M Na_2_HPO_4_) and prepared for their observation under a JEOL® JSM-6380LV microscope (JEOL Ltd., Japan) at the Universidad Austral de Chile (Valdivia, Chile), following the protocol described by Palma-Onetto et al.^57^.

### Structure: light microscope

To identify differential cellular structures in the osmeterium, six larvae were cryoanesthetized at − 4° C. Larvae were dissected and the osmeterium was extracted with the help of ultrathin tweezers and a stereomicroscope Leica® MZ7.5 stereomicroscope (Leica Microsystems, Germany) equipped with a micrometric scale, and then analyzed using the Leica Airlab software. Immediately after dissection, osmeteriums were transferred to the cacodylate buffer (0.2 M, pH 7.3 buffer : 8% glutaraldehyde : distilled water = 2:1:1) for 24 h at 5 °C. Tissues were then washed in a pure 0.1 M cacodylate buffer and postfixed in 2% osmium tetroxide in the same buffer for 2 h. Later, samples were washed with bidistilled water, dehydrated with 50%, 75% and pure ethanol, and then embedded into Spurr resin (standard mixture). Semithin sections were made with a Reichert Ultracut ultramicrotome and stained with methylene blue and studied in an Axioskop Weiss optical microscope (with Sony Cyber-shot digital camera).

### Ultrastructure: transmission electron microscopy

To confirm the glandular property of the cells and to characterize the organelles and cuticle that make the glandular system up, ultrastructural observations were performed. Ultrathin sections were obtained from semithin sections, previously used for observation under the light microscope. Sections were stained with uranyl acetate and lead citrate (standard recipe) and studied in a Jeol 1010 transmission electron microscope.

### Chemical characterization by GC–MS

For chemical analysis of the exocrine gland associated to the osmeterium, ten larvae were anesthetized by cold, and then gently squeezed until the osmeterium was exposed. Once everted, the osmeterium, including the gland, was cut by dissecting scissors. Osmeterium samples were carefully dissected, avoiding samples’ collapse. Rest of hemolymph was dried with an absorbent paper until no visible droplets of hemolymph were observed. Each osmeterium was individually deposited in 70 µL of hexane for 10 min. The hexane solution was then transferred to a new vial and stored at -20 °C till analysis. To understand the origin of secreted VOCs, 15 µL of hemolymph was taken from larval prothorax (after osmeterium removal), and deposited in 70 µL of hexane, following the same procedure as mentioned before. Chemical composition of the osmeterium, hemolymph and reference compounds were analyzed by gas chromatography coupled to mass spectrometry using a GCMS2010 Ultra combination (Shimadzu, Kyoto, Japan). The gas chromatograph was equipped with either a non-polar (SLB-5, 30 m × 0.25 mm, 0.25 μm film; Supelco, Bellefonte, PA, USA) or a polar (Stabilwax, 30 m × 0.32 mm, 0.25 μm film; Restek, Bellefonte, PA, USA) capillary column. For the non-polar column, the oven was programmed from 50 °C (5 min hold) to 270 °C at a rate of 8 °C min^−1^, while for the polar column, the temperature was raised from 50 °C (5 min hold) to 220 °C at a rate of 8 °C min^−1^. The flow rates of the helium carrier gas were 36 cm s^−1^ and 43 cm s^−1^, respectively, and the injector temperature was set to 200 °C in both cases. Mass spectra were acquired at 70 eV with the ion source temperature set to 200 °C. ( +)-Valencene, germacrene B, and β-elemene were purchased from Cayman Chemical (Ann Arbor, MI, USA). Sabinene, β-pinene, and (E)-β-caryophyllene have been obtained from Sigma-Aldrich (St. Louis, MO, USA). Celery seed essential oil was obtained from Essential Hands Aromaterapia (Viña del Mar, Chile). For catalytic hydrogenation, a small amount of palladium (10% on activated charcoal; Merck, Darmstadt, Germany) was added to 100 μL of the extract or 200 μL of a solution (5 μg mL^-1^) of the respective reference compound in hexane. The atmosphere was saturated with H_2_ and the mixture was left to react for 2 h at room temperature, after which it was filtered through a cotton plug and analyzed directly by GC/MS as described above.

### Behavioral assays

To investigate the defensive role of osmeterium VOCs, deterrent effects of osmeterium extracts were evaluated through two-choice assays on the potential predator, the Argentine ant *Linepithema humile*. The Argentine ants are aggressive omnivorous foragers, frequently used as model predators against other arthropods^58–61^, among which caterpillars^62–65^. Moreover, *L. humile* has been previously observed several times predating on dead bodies of third-fourth instar larvae of *B. polydamas archidamas* in the field (González-Teuber, personal observations). Two-choice assay was conducted in glass Petri dish (90 × 20 mm glass Petri dishes), covered with filter paper that were divided into two equal halves by drawing a line through the middle with a grease pencil. Seventy-five µL of osmeterium extract was placed on one side of the Petri dish, whereas at the other side was covered with 75 µL of hexane (as the untreated control). After being anesthetized by cold, ants (N = 7) were carefully placed at the center of each Petri dish, being in direct contact with the filter paper containing both extracts. Ant behavior was observed for 10 min to determine their orientation. A choice was recorded when ants remained on either of the sides for more than 20 s. Ant individuals were used only once, and assays were repeated 20 times. Additionally, a second two-choice assay was developed to evaluate potential differences in deterrent effects between osmeterium and hemolymph extracts. Assays were developed in glass Petri dishes in the same way as described above. Seventy-five µL of each extract was placed at each side of the Petri dish, and ants (N = 10) were placed at the center of them to determine their orientation. Ant individuals were used only once, and assays were repeated 20 times. All assays were conducted in a room at a temperature of 25 °C and 70% relative humidity. Data were analyzed using Wilcoxon signed-rank test with the null hypothesis of equal responses to both choices.

### Supplementary Information

Supplementary Information 1.

Supplementary Video S1.

## Data Availability

Data available on request. Raw data that support the findings of this study are available from the corresponding author, upon reasonable request.
