# Author Correction: Mode of action, chemistry and defensive efficacy of the osmeterium in the caterpillar *Battus polydamas archidamas*

**DOI:** 10.1038/s41598-024-59540-3

**Published:** 2024-04-16

**Authors:** Valeria Palma-Onetto, Jan Bergmann, Marcia González-Teuber

**Affiliations:** 1 Departamento de Química Ambiental, Facultad de Ciencias, Universidad Católica de la Santísima Concepción, Concepción, Chile; 2 Instituto de Química, Facultad de Ciencias, Pontificia Universidad Católica de Valparaíso, Valparaíso, Chile; 3 Departamento de Genética Molecular y Microbiología, Facultad de Ciencias Biológicas, Pontificia Universidad Católica de Chile, Santiago, Chile

Correction to: *Scientific Reports* 10.1038/s41598-023-33390-x, published online 24 April 2023

The original version of this Article contained an error in the Acknowledgements section.

“We thank Alejandra Vergara for her valuable help in the field and in the lab. This work was supported by the ANID-Max Planck Society Grant, MPG190015 (M.G.-T.) and the FONDECYT Grant No. 3210334 (V.P.-O.).”

now reads:

“We thank Alejandra Vergara for her valuable help in the field and in the lab. This work was supported by the ANID-Max Planck Society Grant, MPG190015 (M.G.-T.) and the ANID FONDECYT Postdoc No. 3210334 (V.P.-O.).”

The original Article has been corrected.

