# Supplementary material for: Mode of action, chemistry and defensive efficacy of the osmeterium in the caterpillar Battus polydamas archidamas
Source: Sci Rep. 2023 Apr 24;13:6644. doi: 10.1038/s41598-023-33390-x (PMC10126055; doi:10.1038/s41598-023-33390-x)

**Mode of action, chemistry and defensive efficacy of the osmeterium in *Battus polydamas archidamas***

Palma-Onetto, Valeria^1,*^; Bergmann, Jan^2^; González-Teuber Marcia^1,3,*^

^1^ Departamento de Química Ambiental, Facultad de Ciencias, Universidad Católica de la Santísima Concepción, Concepción, Chile.

^2^ Instituto de Química, Facultad de Ciencias, Pontificia Universidad Católica de Valparaíso, Valparaíso, Chile.

^3^ Departamento de Genética Molecular y Microbiología, Facultad de Ciencias Biológicas, Pontificia Universidad Católica de Chile, Santiago, Chile.

*Corresponding authors:

Valeria Palma-Onetto, vpalma@ucsc.cl

Marcia González-Teuber, mgonzalezt@bio.puc.cl

**SUPPLEMENTARIES**

**Video S1.** Larva of *Battus polydamas archidamas*, curling up its body upon disturbance, everting the osmeterium.

**Figure S1.** Transversal sections of osmeterium arms in *Battus polydamas archidamas*. a) Osmeterium saved, before flipping out. b) Osmeterium everted, exposing the lumen of the secretory system to the exterior. Abbreviations: eg, ellipsoid gland; l, lumen; m, muscle.


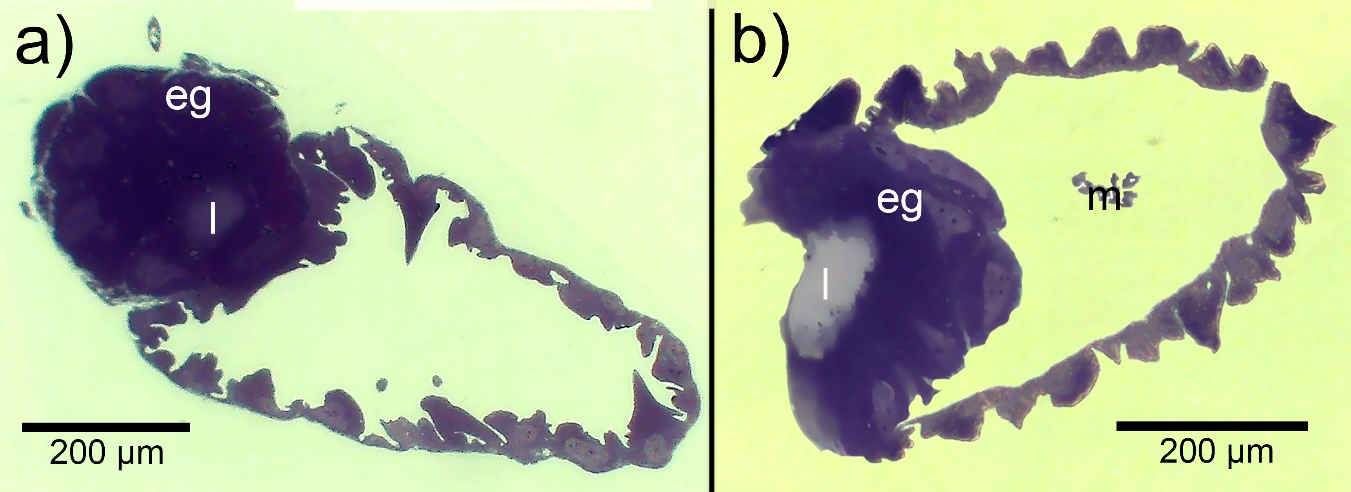


**Figure S2.** Detailed ultrastructure of ellipsoid glands in *Battus polydamas archidamas*. a) Transversal cut of the microvilli at the apex of the cell. b) Apex of the cell, showing abundant mitochondria and secretion vesicles running through the microvilli. c and d) Detail of vesicles in the cytoplasm of the secretory cells. e) Tracheoles located at the base of the ellipsoid gland cells. f) Cuticle at the borders of the gland pore, showing irregular exocuticle. g) Detail of f), note enlarged pore channels ensuring secretion release. Abbreviations: bl, basement lamella; en, endocuticle; ep, epicuticle; ex, exocuticle; l, lumen; m, mitochondria; mv, microvilli; mf, myelin figure; r, ribosomes; rer, rough endoplasmic reticulum; s, secretion; t, tracheola; v, vesicle.


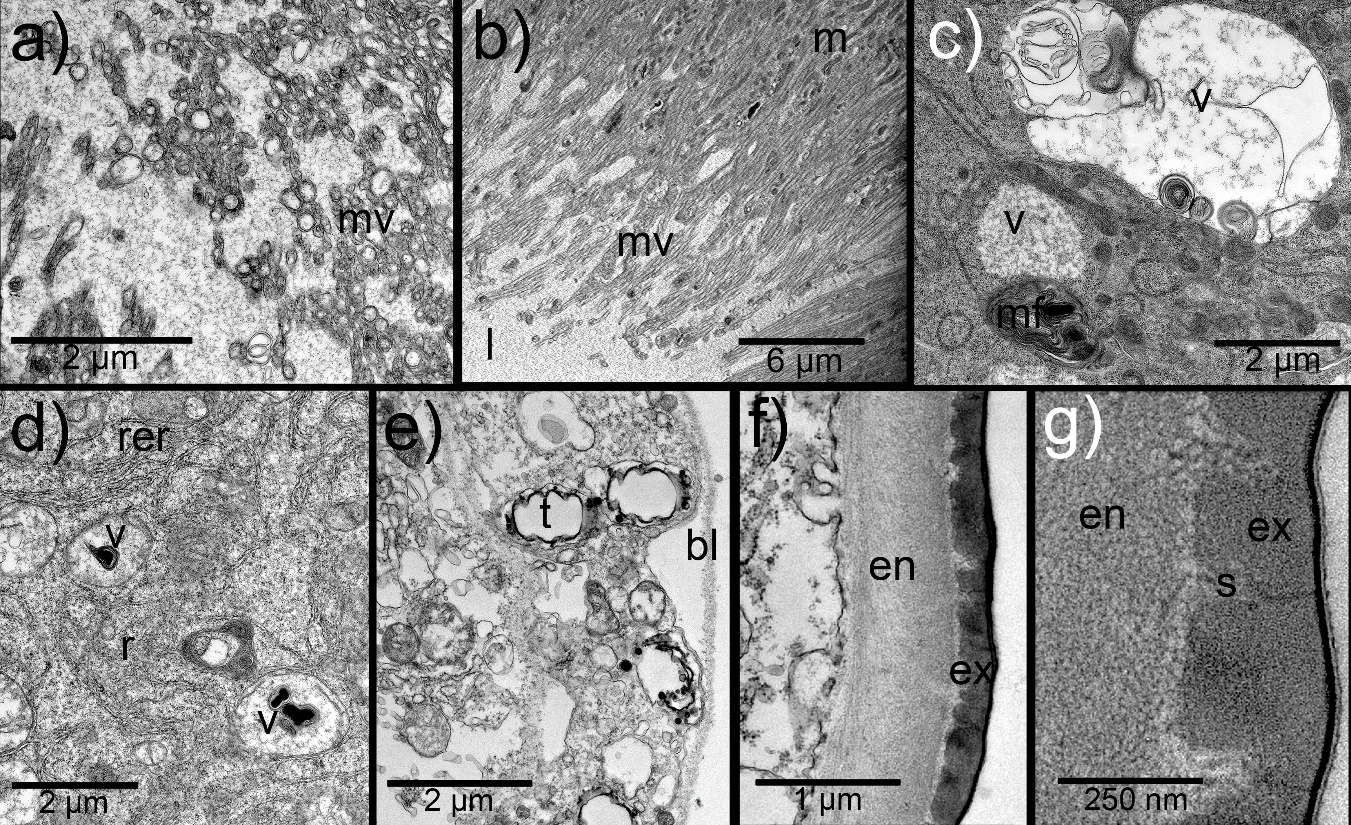


**Figure S3.** Gas chromatograms of a hexane extract of the osmeterial secretion from *Battus polydamas archidamas.* Injector temperature 100 °C (down), injector temperature 200 °C (up).


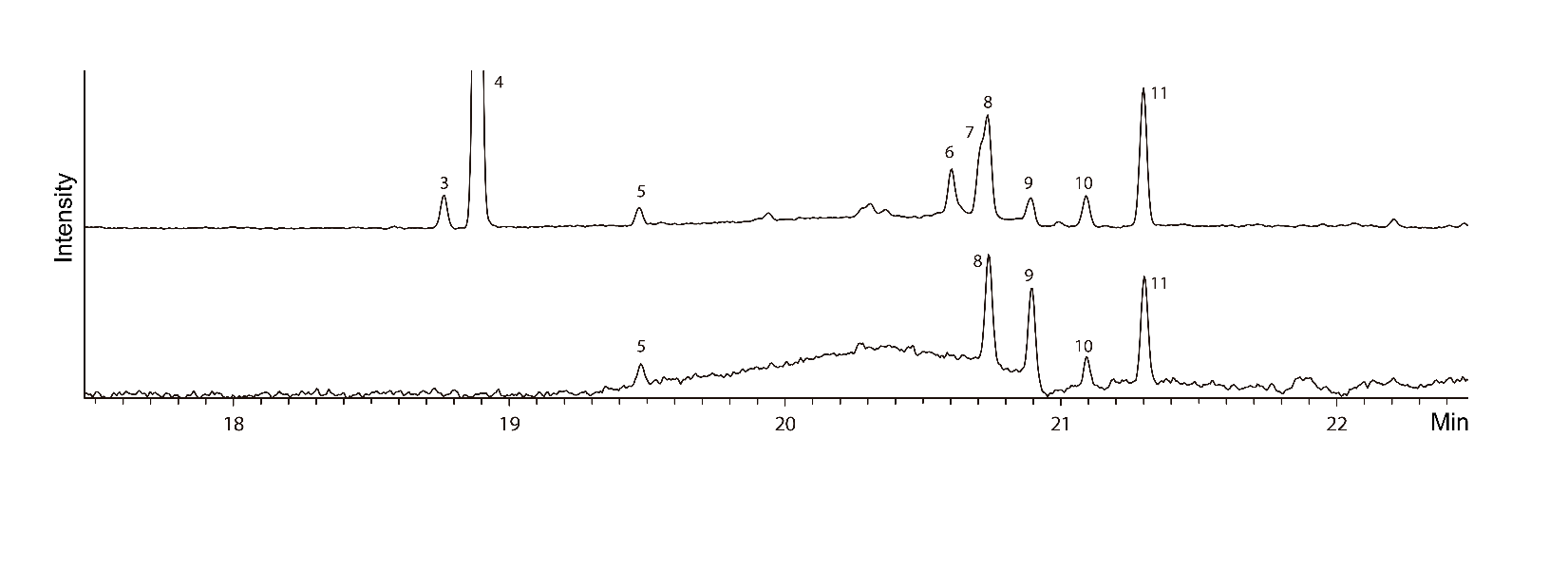

Supplement: Supplementary file 1 — Supplementary Information 1. [file 41598_2023_33390_MOESM1_ESM.docx]
